# It’s a wrap: deriving distinct discoveries with FDR control after a GWAS analysis

**DOI:** 10.1093/bioadv/vbag260

**Published:** 2026-09-04

**Authors:** Benjamin B Chu, Zihuai He, Chiara Sabatti

**Affiliations:** Department of Biomedical Data Science, Stanford School of Medicine, Stanford, CA 94304, USA; Department of Biomedical Data Science, Stanford School of Medicine, Stanford, CA 94304, USA; Department of Neurology and Neurological Sciences, Stanford School of Medicine, Stanford, CA 94304, USA; Quantitative Sciences Unit, Department of Medicine, Stanford School of Medicine, Stanford, CA 94304, USA; Department of Biomedical Data Science, Stanford School of Medicine, Stanford, CA 94304, USA; Department of Statistics, Stanford University, Stanford, CA 94304, USA

## Abstract

**Motivation:**

The standard analysis pipeline for genome-wide association studies (GWAS) is based on marginal tests of association. These are computationally convenient and portable, but the discoveries are not immediately interpretable and require post-processing such as “clumping” and “fine mapping.” An interesting alternative is provided by conditional independence hypotheses: their rejections lead to distinct signals across the genome, accounting for measured confounders and pointing to separate causal pathways. Recent work has shown how summary statistics resulting from the standard marginal GWAS can be used as input to test conditional independence hypotheses while controlling the false discovery rate (FDR). We previously developed a pipeline tailored to European genomes.

**Results:**

Here we introduce and release new software (solveblock) extending this capability to a much richer collection of studies. Given a set of genotyped samples, or a reference dataset, the new pipeline efficiently estimates the high-dimensional correlation matrices that describe dependencies across the genome, making rather common sparsity assumptions. Taking this sample-specific estimate as input, the software identifies groups of genetic variants that are highly correlated and uses them to define an appropriate resolution for conditional independence hypotheses. Finally, we compute the distribution for the exchangeable negative controls necessary to test these hypotheses. Simulations based on five UK Biobank sub-populations, illustrate the method’s FDR control. The analysis of 26 phenotypes of varying polygenicity in British individuals, results in ≈19 additional discoveries, compared to standard marginal association testing.

**Availability and implementation:**

Our code, precompiled software, and processed files for these five sub-populations are openly shared.

## 1 Introduction

The knockoff filter (Candès *et al.* 2018) is a variable selection framework that tests conditional independence hypotheses while controlling the false discovery rate (FDR) (Benjamini and Hochberg 1995). The approach is particularly appealing in genome-wide association studies (GWAS), where it leads to interpretable discoveries that do not require post-processing and are closely tracking causal variants (Sesia *et al.* 2020). A successful implementation of this strategy models the distribution of genotypes using hidden-Markov models (HMM) (Sesia *et al.* 2019, 2021), in line with the genetic literature. Prior to a knockoff analysis, however, this approach requires phasing the genotypes, estimating parameters of an HMM, and determining identical-by-descent segments. All these steps are computationally intensive, despite recent advances (Chu *et al.* 2021, Hofmeister *et al.* 2023, Browning and Browning 2024), and require reliance on a collection of external tools not optimized for this specific goal. As a result, a knockoff analysis of GWAS data based on HMMs is still much more involved than the traditional GWAS pipeline.

An alternative path to testing conditional hypotheses across the genome has started to emerge with the realization that an approximate knockoffs analysis can be carried out working not with individual level data, but with summary statistics capturing the correlation between the outcome and the explanatory variables (He *et al.* 2022, Chen *et al.* 2024). This procedure, known as GhostKnockoffs, takes as input GWAS summary statistics (e.g. *P*-value and direction of effect) and can therefore be run without changing any of the standard preprocessing steps. Empirical studies (Qi *et al.* 2023) show a particularly appealing characteristic of this pipeline: the input can be any testing statistic that is appropriate for the data, such as those derived from linear mixed models that account for relatedness. In He *et al.* (2026), the re-analyses of 67 European GWASes with this framework led to greater power and improved precision, as compared with the results of the original analyses.

While the GhostKnockoffs approach has the potential for a more straightforward harmonization with the standard GWAS pipeline, the necessity of modeling the distribution of genotypes and calculating the derived knockoff distribution remains. Since this approach relies on matching the first two moments, crucial elements are (i) the matrix of correlations between allelic counts of different SNPs and (ii) the sampling of powerful second-order knockoffs (Chu *et al.* 2024). The authors of He *et al.* (2026) distributed software (CIT-Lasso) that takes as input GWAS summary statistics and parameters that characterize the knockoff sampling distribution (a, b). To facilitate users, precomputed parameters appropriate for a European population, derived from LD matrices calculated on data from the UK Biobank (Sudlow *et al.* 2015, Pan-UKB team 2020), were also made available on Zenodo (European Organization For Nuclear Research and OpenAIRE 2013).

In this work, we extend the access to a computationally convenient and reliable knockoff analysis to a much richer set of studies. Given that GhostKnockoff can be used as a “wrapper” around any standard pipeline, what remains is to provide users with a way of computing the parameters that characterize the knockoff sampling distribution (a, b) that are appropriate for their specific sample. The software we introduce assumes that individual-level genotype data is available. This can be from the sample under study or an appropriate reference set. Taking this as input, it (i) estimates high-dimensional LD matrices appropriate for the target sample, (ii) identifies groups of SNPs in high disequilibrium that will be the object of conditional inference, (iii) approximates the dependency structure across these groups, and (iv) computes the parameters of the joint variance-covariance matrix of the original variables and knockoffs to maximize power. Note that if an adequate LD matrix is available (instead of individual level data), step (i) can be skipped. One of the purposes of the released software is to facilitate step (i) when this is not the case.

Thanks to the solveblock binary executable, users can construct the bundle of files containing LD-related information and necessary for GhostKnockoff analysis directly from individual-level data stored in VCF (Danecek *et al.* 2011) or binary PLINK (Purcell *et al.* 2007) format. To run a genome-wide knockoff-based conditional independence testing, users need to (i) run any standard GWAS pipeline to obtain marginal Z-scores, (ii) run the solveblock executable to obtain parameters for knockoff construction, and (iii) input the results of these two first steps into CIT-Lasso. Users have the option to (iv) share the output of solveblock by uploading it to services such as Zenodo (European Organization For Nuclear Research and OpenAIRE 2013). Simulation studies and real-data examples illustrate the speed, enhanced power, and FDR control of knockoff-based inference enabled by this pipeline.

## 2 Results

### 2.1 The software

Figure 1 gives a broad overview of the analysis pipeline, highlighting the steps executed by solveblock. Details on each step are available in Section 5, available as supplementary data at *Bioinformatics Advances* online. The software is written in Julia (Bezanson *et al.* 2017) and distributed freely on GitHub under the broader GhostKnockoff framework: github.com/biona001/CITLasso. For users who do not wish to install Julia, it is also compiled into a standalone executable.

**Figure 1:**
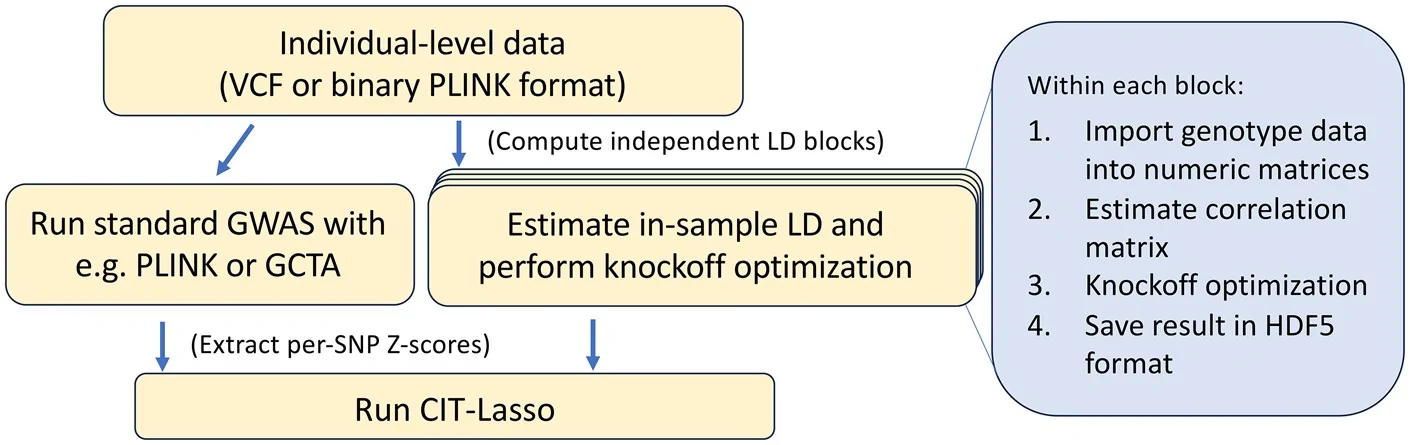
GhostKnockoff analysis pipeline. The solveblock executable carries out the tasks identified in the blue box.

To illustrate the capabilities of solveblock, we are going to leverage simulations and real data analysis. Both are based on data obtained from the UK Biobank. More details on data preprocessing can be found in Section 1.1, available as supplementary data at *Bioinformatics Advances* online. Applying solveblock on the individual-level genotypes of samples identified as British, Indian, Caribbean, African, and Chinese gives us information on LD patterns that can be used to identify different groups of SNPs and generate efficient knockoffs. Results are summarized in Table 1, available as supplementary data at *Bioinformatics Advances* online.

### 2.2 FDR control: simulations

The results of 200 independent simulations for various populations demonstrate valid (group) FDR control, as depicted in Figure 2A. The procedure consists of running solveblock + CIT-Lasso with respect to different UKB sub-populations. The variation in power across populations is a consequence of variable sample size, as phenotypes are generated from the same distribution. Details can be found in Section 3, available as supplementary data at *Bioinformatics Advances* online.

**Figure 2:**
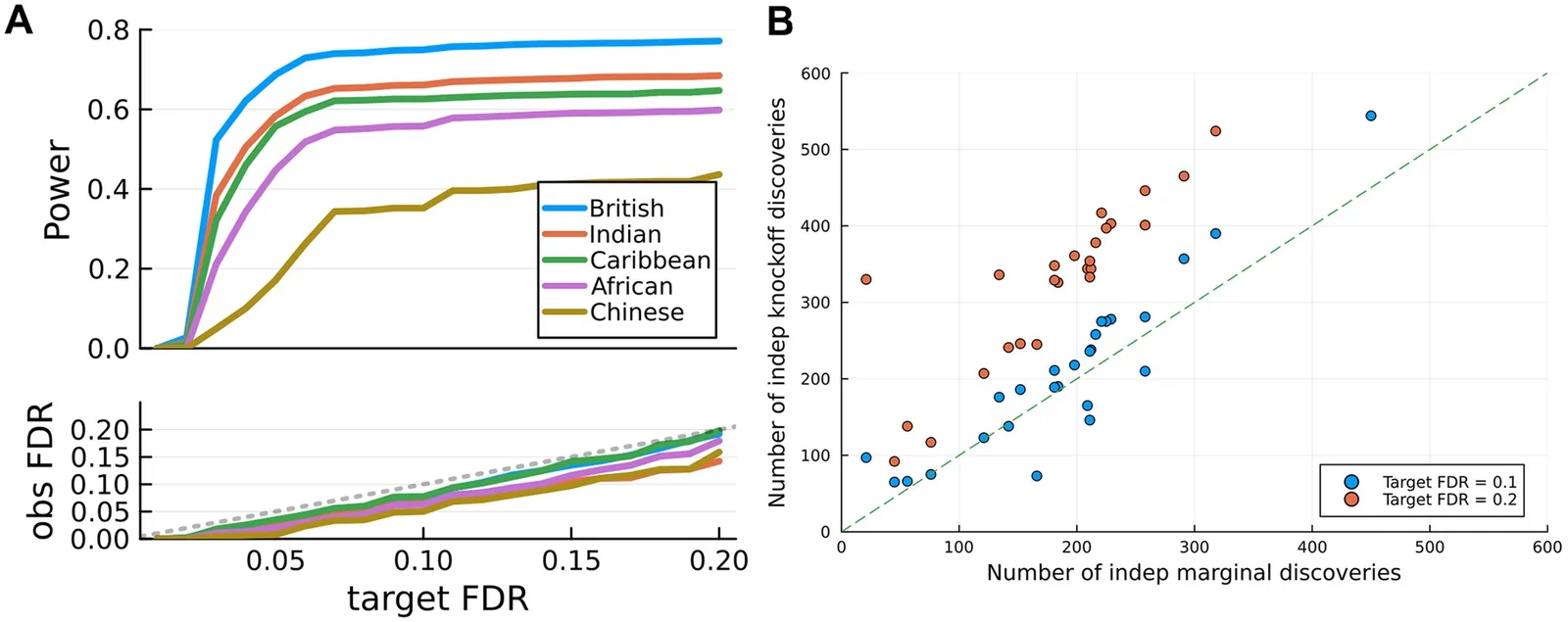
Simulation results and UK Biobank discoveries. (A) Average power and false discovery rate across 200 simulations. The British subset contains only unrelated individuals; related samples are included for the other populations. (B) Number of independent discoveries from GhostKnockoff compared with a conventional marginal genome-wide association study. Each dot represents 1 of 26 phenotypes, each analyzed in <15 minutes on a single CPU. Dots above the dashed line indicate more signals from the knockoff analysis. Details are provided in Table 2, available as supplementary data at *Bioinformatics Advances* online.

### 2.3 Exploring power: analysis of 26 UKB phenotypes

Here we analyzed 26 continuous phenotypes based on the British samples of UKB. The goal of this section is to (i) demonstrate that the LD files created by solveblock can be used directly for a GhostKnockoff analysis and (ii) demonstrate the scalability of GhostKnockoff on biobank-scale datasets (each phenotype took <15 minutes to run on a single CPU), and (iii) demonstrate the enhanced power of GhostKnockoff in locus discovery compared to marginal analysis methods. For details of the analysis, see Section 1.2, available as supplementary data at *Bioinformatics Advances* online.

Figure 2B compares the number of independent discoveries from knockoff-based analyses to that from conventional marginal GWAS with the Bonferroni threshold set to $5\times 10^{-8}$. While each knockoff discovery is by construction distinct, for ease of comparison, 2 discoveries (coming from marginal or knockoff analyses) are considered *independent* if they are at least 1 Mb apart. Based on this criterion, GhostKnockoff analyses found 19.0 (149.1) additional independent discoveries compared to marginal testing when the target FDR is 10% (20%). Table 2 in the Supplement provides more details, available as supplementary data at *Bioinformatics Advances* online. Notably, GhostKnockoff discovered more signals in 20 out of 26 traits at a given target FDR of 10%, and each phenotype took 15 minutes or less to run on a single CPU core on the cloud. These results showcase our method’s enhanced power and computational efficiency for locus discovery.

## 3 Discussion

The goal of this paper is two-fold. First, we introduce the solveblock executable. When combined with the accompanying software CIT-Lasso (He *et al.* 2026), this can be used to perform an end-to-end GWAS analysis using a knockoff perspective while adhering to a standard GWAS pipeline. We found that this approach is scalable to biobank-sized data and controls the FDR, while delivering enhanced power.

The second goal of this paper is to provide a platform for and encourage data exchange. Prior to this work, precomputed LD parameters necessary for GhostKnockoff analyses were available only for European samples. We now provide these for Indian, Caribbean, African, and Chinese samples, with the caveat that the parameters have been constructed from the fairly small sample sizes available in the UK Biobank. More impactfully, the solveblock executable introduced in this paper will allow investigators to generate parameters that reflect the LD in their specific samples. By construction, the output files are small in size (Table 1, available as supplementary data at *Bioinformatics Advances* online), do not contain private information, and are therefore easy to share. For example, the results can be easily uploaded to Zenodo (European Organization For Nuclear Research and OpenAIRE 2013). If just a small fraction of our users are open to data-sharing, CIT-Lasso will become exponentially more versatile.

Further extensions are also planned. First, we note that all GhostKnockoff computation can feasibly be carried out entirely on the cloud. We then envision the construction of a utility similar to Michigan’s imputation server (Das *et al.* 2016): users would upload GWAS summary statistics, select the desired LD panel to use, and later download the GhostKnockoff discoveries. This bypasses the need to distribute processed LD files and avoids headaches with software installation.

Second, it is of interest to extend the ghost-knockoff methodology to non-SNP-array data, such as those generated from high-coverage whole-genome sequencing or low-pass imputation-based data (Boltz *et al.* 2026). Such dense genotyping technologies are expected to become increasingly affordable, eventually leading to a higher adoption rate than array-based data in genetic studies. This data differs from traditional SNP genotypes in two regards. First, it contains significantly more SNPs per independent window (often exceeding $10^{5}$ variants). A number of these variants are expected to be in high linkage disequilibrium, so that using group-based inference will be crucial. To avoid excessively increasing the computational burden, additional preprocessing steps and computational innovation for GhostKnockoff sampling (He *et al.* 2026) will be required. Secondly, WGS allows the evaluation of the effect of rare variant analysis (e.g. MAF <1%). Knockoff methods based on individual-level data (Sesia *et al.* 2021) or summary statistics (He *et al.* 2021) have been developed to analyze this type of variant, and they can be integrated into the solveblock workflow. Specifically, rare variants often require different grouping strategies or the use of burden/SKAT-like statistics within the knockoff framework to maintain power.

In closing, we note that when working with multi-ancestry biobanks like All of Us (All of Us Research Program Investigators 2019) and the Million Veteran Project (Gaziano *et al.* 2016), a common question is whether to perform the analysis separately by ancestry or using a pooled approach. In the context of GhostKnockoff, we recommend ancestry-specific analysis if summary statistics and LD panels are available for the different groups. If it is not easy to identify distinct population subgroups within a study solveblock can be applied on individual level data to model the pooled sample’s LD structure, ensuring FDR control.

We raise these research questions to stimulate discussion and encourage participation from the broader community.

## Supplementary Material

vbag260_Supplementary_Data

## Data Availability

**Project home page**: https://github.com/biona001/CITLasso. **Supported operating systems**: Linux and macOS. **Programming language**: Written in Julia (Bezanson *et al.* 2017) but the program is compiled into standalone executables. **License**: MIT. **Reproducibility scripts**: The code used to run the simulated experiments and real-data analyses can be found in https://github.com/biona001/solveblock-reproducibility.
