# Supplementary material for: It’s a wrap: deriving distinct discoveries with FDR control after a GWAS analysis
Source: Bioinform Adv. 2026 Sep 4;6(1):vbag260. doi: 10.1093/bioadv/vbag260 (PMC13676318; doi:10.1093/bioadv/vbag260)
Supplement: Supplementary file 1 [file vbag260_supplementary_data.pdf]

Supplement to: It's a wrap: deriving distinct discoveries with FDR control after a GWAS analysis

Benjamin B. Chu,[1] Zihuai He[1,2,3] and Chiara Sabatti[1,4,*]

[1]Department of Biomedical Data Science, Stanford School of Medicine
[2]Department of Neurology and Neurological Sciences, Stanford School of Medicine
[3]Quantitative Sciences Unit, Department of Medicine, Stanford School of Medicine
[4]Department of Statistics, Stanford University

[*]Corresponding author. sabatti@stanford.edu

Abstract

This document provides supplementary data-processing details, simulation settings, computational benchmarks, phenotype-level results, and implementation methods for the accompanying article.

1. UK Biobank data

1.1. Quality Control

The second release of the UK Biobank (UKB) data (Sudlow et al., 2015) contains $\sim 500,000$ samples of primarily European descent and $\sim 800,000$ SNPs without imputation. Following (Sesia et al., 2020), for British samples, we kept only 1 sample from each of the 60,169 familial groups. This resulted in $n = 306,629$ unrelated British samples. Next, we also created different sub-populations based on samples identified as Indian ($n = 5951$), Caribbean ($n = 4517$), African ($n = 3394$), and Chinese ($n = 1574$). For these non-British sub-populations, we kept related samples to mimic cryptic relatedness in downstream simulations. For all five UKB sub-populations, we excluded non-biallelic SNPs and SNPs with minor allele frequency (MAF) < 0.01 and Hardy-Weinburg equilibrium p-values $< 10^{-6}$. Finally, for our real-data analysis, we chose 26 continuous and commonly measured phenotypes (see Table 2) as targets of analyses. Samples that cannot be matched to phenotype file by ID or whose phenotypes contain missing values were also excluded.

Non-genetic covariates. Following Pan-UKB (Pan-UKB team, 2020), we included the following covariates for LD adjustments: sex, age, age^2, sex×age, sex×age^2, and the top 10 principal components precomputed by the UK-Biobank. These covariates form the matrix $\mathbf{C}$ in equation (2).

1.2. Statistical analysis

Here we provide additional details on the UK Biobank analysis (British samples) as featured in our main paper. For each phenotype, we first conduct a standard marginal analysis using the score statistic after regressing out the effect of covariates from the phenotype vector. Mathematically, let $\mathbf{y}$ be the original phenotype vector, $\mathbf{C}$ be the matrix of covariates, and $\mathbf{X}$ the genotype matrix that is column standardized to mean 0 variance 1. We first compute $\tilde{\mathbf{y}} = (\mathbf{I} - \mathbf{C}(\mathbf{C}^t\mathbf{C})^{-1}\mathbf{C}^t)\mathbf{y}$ and standardize it to mean 0 variance 1. Then we compute $\mathbf{z} = \mathbf{X}^t\tilde{\mathbf{y}}/\sqrt{n}$ where $n = 306,629$ British samples. The resulting $\mathbf{z}$ in combination with the output of `solveblock` are then used as inputs for `CIT-Lasso` (He et al., 2026).

2. Analysis of LD and `solveblock` output

Our first goal is to construct the necessary LD files for various subpopulations of the UKB, to enable knockoff-based analysis and simulations on these populations. To do so, we first applied the `snp_ldsplit` functionality of `bigsnpr` package (Privé, 2022) to each chromosome of each subpopulation of UKB separately. We used the parameters `thr_r2` = 0.01, `max_r2` = 0.3, `min_size` = 500, and `max_size` = {1000, 1500, 3000, 6000, 10000}. Given block boundaries in each subpopulation, we applied our `solveblock` executable directly on the individual-level genotypes.

© The Author(s) . Copyright and licence statements to be updated by the publisher during production.

2.1. Summary of distributed LD files

The output files for each subpopulation are a few dozen gigabytes in size. Table 1 summarizes the final number of blocks and disk space needed to store the resulting LD files for each sub-population.

Data	Sample size (n)	Number of blocks (b)	Disk space (GB)	Number of SNPs (p)*
British	306,629	636	20.0	551,597
Indian	5,951	615	13.0	463,739
Caribbean	4,517	489	13.0	365,725
African	3,394	513	9.4	334,004
Chinese	1,574	505	7.4	310,868

Table 1 Summary of distributed linkage disequilibrium files. Results after applying the `snp_ldsplit` function of the `bigsnpr` package to individual-level UK Biobank data. *Number of SNPs with MAF $\geq$ 0.01.

2.2. Computational efficiency

Because we partitioned the genome into independent blocks (Equation (1)), `solveblock` can process each block independently. We suggest running each block in parallel, e.g., via cluster jobs, to minimize total wall-clock time.

To evaluate the computational requirements of `solveblock`, we benchmarked each of the 52 blocks on chromosome 1 for the British population ($n = 306,629$). We chose chromosome 1 as it is the largest chromosome and should be representative of the maximum computational load. On average, each job completed in approximately 48 seconds (maximum 106 seconds) with peak memory usage under 11 GB. For smaller sub-populations, jobs typically completed in under 10 seconds with peak RAM under 5 GB.

3. Simulations: FDR control

In this section, we include the details for (group) FDR vs power simulations, as featured in our main paper. We restrict our attention to SNPs falling on chromosome 22, totaling $p = 8878$ SNPs. In all simulations we column-standardize $\mathbf{X}$ and simulate the response as $\mathbf{y} = \mathbf{X}\boldsymbol{\beta} + \mathcal{N}(\mathbf{0}, 3\mathbf{I}_{n\times n})$ where $\boldsymbol{\beta}$ contain $k = 10$ non-zero effects chosen randomly and effect sizes drawn from $\mathcal{N}(0, 0.5)$. The narrow sense heritability for these simulations is approximately 0.25. After regressing out the effect of covariates, we standardize the phenotype.

Marginal Z-scores were computed in two ways. For the British samples, where related individuals are removed, we compute marginal Z-scores by the score statistic $\mathbf{z} = \mathbf{X}^t\mathbf{y}/\sqrt{n}$ where $\mathbf{y}$ is standardized. For other populations in the UKB, which contain related samples and potential continental admixture, we use the linear mixed model software GCTA (Jiang et al., 2021) to compute p-values, and transformed them back into Z-scores. Once LD files are estimated from genotypes, and Z-scores evaluated, they are used as the only inputs used for `CIT-Lasso` for evaluating power and (group) FDR.

4. Table formatted result for UK Biobank analysis of 26 phenotypes

Table 2 contain the number of independent discoveries in a marginal analysis vs GhostKnockoff analysis, with target FDR level set to 10% and 20%. Runtimes are also provided.

Phenotype	# marginal	GK FDR 10%	GK FDR 20%	GK runtime (s)
Height	450	544	722	533
Weight	258	210	401	540
Waist circumference	166	73	245	540
Body mass index	209	165	344	760
Body fat mass	211	146	333	485
Diastolic blood pressure	121	123	207	746
Systolic blood pressure	142	138	241	698
Whitecell	184	190	326	756
Redcell	258	281	446	766
Haemoglobin	198	218	361	768
Haematocrit	181	211	348	717
Corpuscular volume	229	278	403	754
Corpuscular haemoglobin	212	238	344	835
Redcell width	152	186	246	759
Platelet	318	390	524	540
Platelet volume	291	357	465	805
Platelet width	216	258	378	693
Lymphocyte	56	66	138	711
Monocyte	134	176	336	705
Neutrophill	211	236	354	824
Eosinophill	181	189	329	725
Basophill	45	65	92	700
Nucleated redcell	21	97	330	484
Reticulocyte	76	75	330	485
Reticulocyte volume	225	275	397	485
Spheredcell volume	221	275	417	492

Table 2 Independent discoveries for 26 UK Biobank phenotypes. Results are shown for the knockoff framework and conventional marginal hypothesis testing. GK stands for ghost-knockoff (using the `CIT-Lasso` implementation), and FDR stands for false discovery rate. The target false discovery rate is 10% or 20%. Runtime corresponds to `CIT-Lasso` and excludes `solveblock`, which only needs to be run once for a population of interest. Peak memory usage for all phenotypes was less than 16.0 GB.

5. Methods

We assume that the user has available a set of genotypes $\mathbf{X}$ on n individuals (rows) and p SNPs (columns) as well as summary statistics on the association between these same SNPs and a phenotype of interest. The genotypes might have been obtained as part of the study that lead to the association summary statistics, or belong to an appropriate reference panel. We note that when individual level data on genotype and phenotype is available, one can conduct a knockoff-based analysis by generating knockoffs $\tilde{\mathbf{X}}$ using HMMs distribution (Sesia et al., 2019, 2020, 2021). While there are many robustness advantages of this approach, it remains more computationally involved, and it is more difficult to harmonize with analysis pipelines that might have been pre-established, as it is often the case in the context of consortia. In what follows, we work with a GhostKnockoff (Chen et al., 2024) pipeline (He et al., 2026).

Preprocessing steps

For GWAS data with $p \approx 10^6$, it is common to assume that the variance-covariance matrix $\mathbf{\Sigma}$ for the SNPs has a block-diagonal form (Berisa and Pickrell, 2016; Privé, 2022):

$$\hat{\mathbf{\Sigma}} = \begin{bmatrix} \hat{\mathbf{\Sigma}}_1 & & & \\ & \hat{\mathbf{\Sigma}}_2 & & \\ & & \ddots & \\ & & & \hat{\mathbf{\Sigma}}_b \end{bmatrix} \in \mathbb{R}^{p \times p}, \tag{1}$$

effectively assuming that SNPs in different blocks are independent. Defining blocks in correspondence to different chromosomes is certainly natural. However, in order to reduce computational burden, researchers typically consider blocks of smaller sizes. It is important to recall that if the block-wise independence reflected in (1) is not (at least approximately) satisfied, the knockoff analysis resulting from the pipeline we are about to describe will not have FDR control.

The block boundaries can be computed efficiently using the `snp_ldsplit` function of the `bigsnpr` package (Privé, 2022) and need to be identified before `solveblock` can be run. Empirically, working in a variety of populations, we found that b on the order of $10^2 \sim 10^3$ is sufficient to partition the human genome based on the default objective provided in `bigsnpr`, see Table 1. Specifically, we used `max_size` parameters ranging from 1,000 to 10,000 SNPs. Choosing a larger `max_size` allows for capturing longer-range LD at the expense of increased computational time and memory during the `solveblock` step. For most datasets, a `max_size` between 2,000 and 5,000 provides a good balance between computational efficiency and capturing sufficient linkage disequilibrium for valid FDR control. To obtain reliable estimates to genetic correlation, we recommend that only SNPs with minor allele frequency ≥ 0.01 should contribute to LD block estimation. Note that, while

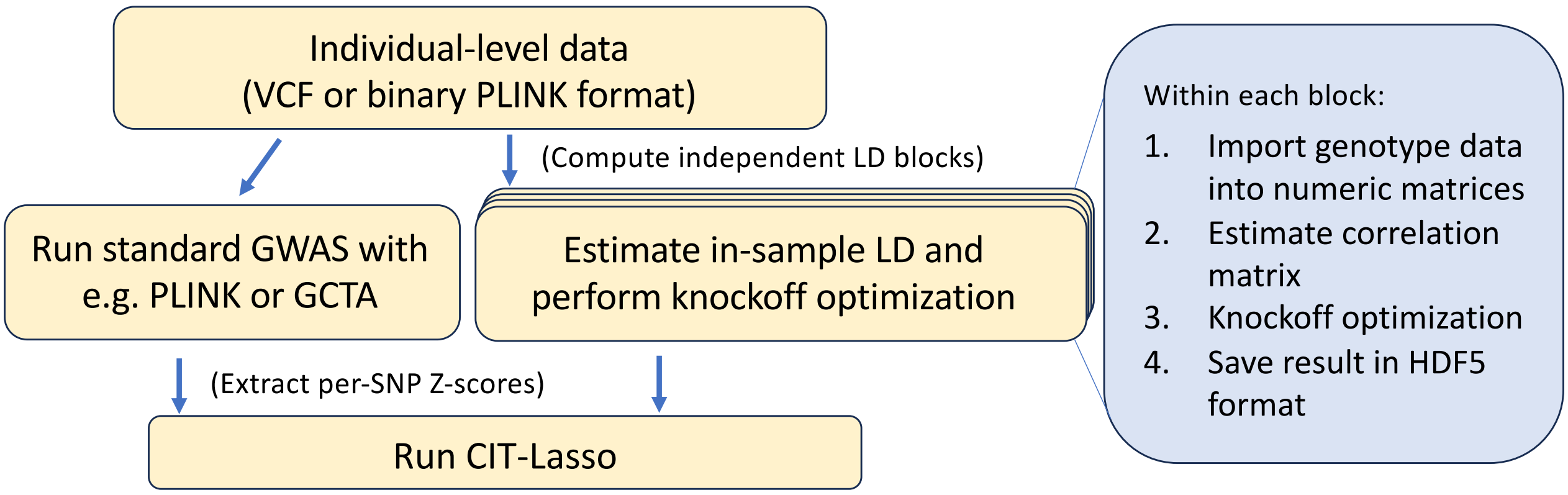

Figure 1 GhostKnockoff analysis pipeline. The sequence of events performed by the `solveblock` executable is outlined in the blue box.

more numerous than the chromosomes, these blocks include a large number of SNPs, so that they can be used to represent a fairly extensive linkage disequilibrium.

Within each block $i = 1, ..., b$ in equation (1), `solveblock` will perform 4 main steps, summarized in Figure 1, and described in the following.

5.1. Import genotype data into computer memory

We import the genotype data into double-precision matrices $x_{ij} \in \{0, 1, 2, \text{missing}\}$ using the `OpenMendel` (Zhou et al., 2020) modules `SnpArrays.jl` or `VCFTools.jl`. This step requires $8np_i$ bytes of computer RAM, which is memory intensive if p_i (number of variants in block i) is extremely large. Typically, however, the block-diagonal approximation (1) implies p_i is generally on the order of 10^3, which can be handled efficiently.

For VCF inputs, our convention is to convert each `ALT` allele into numeric value of 1. With binary PLINK inputs, all `A2` alleles are converted into 1 even if it is the major allele (note this is equivalent to using `---keep-allele-order` within `PLINK 1.9` software). Multi-allelic SNPs should be split prior to analysis. Missing genotypes are automatically imputed by column mean.

5.2. Estimation of correlation matrix

Within region $i \in \{1, ..., b\}$, let $\mathbf{X}_i \in \mathbb{R}^{n \times p_i}$ denote the column-standardized genotype matrix. Our next goal is to estimate a correlation matrix. Note that the pre-processing step of identifying block boundaries technically is based on measures of correlation, implicitly used by `snp_ldsplit` function of `bigsnpr` package. Once the boundaries are identified, however, we explicitly estimate a correlation matrix (denoted by $\hat{\mathbf{\Sigma}}_i$) that will be used in the GhostKnockoff pipeline. We adopted the same LD estimation routine of Pan-UKB (Pan-UKB team, 2020) which we found to perform well (Chu et al., 2024). Letting $\mathbf{C}$ denote a matrix of covariates (sex, age, etc), we compute:

$$
\begin{aligned}
\mathbf{M}_c &= \mathbf{I} - \mathbf{C}(\mathbf{C}^t\mathbf{C})^{-1}\mathbf{C}^t \quad \text{(the residual-maker matrix)} \\
\mathbf{X}_{adj} &= \mathbf{M}_c\mathbf{X}_i \quad \text{(regressing out effect of covariates)} \\
\hat{\mathbf{\Sigma}}_i &= \frac{\mathbf{X}_{adj}^t\mathbf{X}_{adj}}{n}
\end{aligned} \tag{2}
$$

For numerical stability, we set the minimum eigenvalue of $\hat{\mathbf{\Sigma}}_i$ to be 0.00001 and scaled the final result so the diagonal entries are exactly 1. (Inputting the matrix of covariates $\mathbf{C}$ is optional).

5.3. Parameters for group-knockoffs generation

Our next step is to calculate the parameters that describe the variance-covariance matrix of the original variables and knockoffs. For each block $\mathbf{\Sigma}_i$, this includes 3 main steps: (1) identify groups of highly correlated SNPs that are going to be the basis of inference, (2) approximate the joint distribution of all SNPs using a conditional independence model in which only a subset of "key variables" within each group are driver of the correlations across groups, and (3) find the variance-covariance matrix for $(X, \tilde{X})$ that satisfies the exchangeability constrains while maximizing detection power by maximizing the entropy criteria (Gimenez and Zou, 2019). The motivation behind all of these steps and the algorithms required to carry them out are described elsewhere (Chu et al., 2024). We summarize them below for completeness.

Following (Chu et al., 2024), for each $\hat{\mathbf{\Sigma}}_i \in \mathbb{R}^{p_i \times p_i}$, we define non-overlapping groups via average-linkage hierarchical clustering with correlation cutoff 0.5. Given group boundaries, we then apply the best subset selection algorithm of (Chu et al., 2024) (Algorithm A2 in the supplement) with $c = 0.5$ to identify *group-key* variables within each group. Intuitively, the group-key variables are a subset of $\{1, ..., p_i\}$ that drive the dependence across groups. The sampling of knockoffs, then, can be carried out by sampling knockoffs for these key variables and then, within each groups, knockoffs for the remaining variables conditional of these. Let R denote the set of group-key variables, C denote

its complement, and $(\hat{\mathbf{\Sigma}}_i)_{(R,R)}$ the rows and columns of $\hat{\mathbf{\Sigma}}_i$ corresponding to variables in R. Knockoff optimization proceeds by solving the maximum entropy (Gimenez and Zou, 2019) objective

$$\max_{\mathbf{S}_i} \log\det\left(\frac{m+1}{m}(\hat{\mathbf{\Sigma}}_i)_{(R,R)} - \mathbf{S}_i\right) + m\log\det(\mathbf{S}_i) \quad \text{subject to } \begin{cases} \frac{m+1}{m}(\hat{\mathbf{\Sigma}}_i)_{(R,R)} - \mathbf{S}_i \succeq 0 \\ \mathbf{S}_i \succeq 0 \end{cases} \tag{3}$$

where $m = 5$ is the number of knockoffs to generate per feature and $\mathbf{S}_i \succeq 0$ means $\mathbf{S}_i$ is positive semi-definite. We used the coordinate descent algorithm implemented in `Knockoffs.jl` (Chu et al., 2024) to solve equation (3). Once $\mathbf{S}_i$ is determined, the $p_i \times p_i$ matrix

$$\begin{aligned} \mathbf{D}_i &= \begin{pmatrix} \mathbf{S}_i & \mathbf{S}_i(\hat{\mathbf{\Sigma}}_i)^{-1}_{(R,R)}(\hat{\mathbf{\Sigma}}_i)_{(R,C)} \\ (\hat{\mathbf{\Sigma}}_i)_{(C,R)}(\hat{\mathbf{\Sigma}}_i)^{-1}_{(R,R)}\mathbf{S}_i & \mathbf{D}_{i,22} \end{pmatrix} \text{ where} \\ \mathbf{D}_{i,22} &= (\hat{\mathbf{\Sigma}}_i)_{(C,C)} - (\hat{\mathbf{\Sigma}}_i)_{(C,R)}(\hat{\mathbf{\Sigma}}_i)^{-1}_{(R,R)}(\hat{\mathbf{\Sigma}}_i)_{(R,C)} + (\hat{\mathbf{\Sigma}}_i)_{(C,R)}(\hat{\mathbf{\Sigma}}_i)^{-1}_{(R,R)}\mathbf{S}_i(\hat{\mathbf{\Sigma}}_i)^{-1}_{(R,R)}(\hat{\mathbf{\Sigma}}_i)_{(R,C)} \end{aligned}$$

is the solution to the maximum entropy objective for the full $\hat{\mathbf{\Sigma}}_i \in \mathbb{R}^{p_i \times p_i}$. In particular, $\mathbf{D}_i \succeq 0$ satisfies the constraints $\frac{m+1}{m}\hat{\mathbf{\Sigma}}_i - \mathbf{D}_i \succeq 0$ and can be used for ghost-knockoff sampling (He et al., 2021).

5.4. Output in HDF5 format

Up until this point, we have assembled the reduced data:

- $\hat{\mathbf{\Sigma}}_1, ..., \hat{\mathbf{\Sigma}}_b$: correlation matrices for each block
- $\mathbf{S}_1, ..., \mathbf{S}_b$: knockoff optimization matrices for each block, carried out on the group-key variables
- $\mathbf{D}_1, ..., \mathbf{D}_b$: knockoff sampling matrices for each block satisfying $\frac{m+1}{m}\hat{\mathbf{\Sigma}}_i - \mathbf{D}_i \succeq 0$.
- Group membership vector for each block $i = \{1, ..., b\}$
- Group-key variables for each block $i = \{1, ..., b\}$

We will save this ensemble of data in HDF5 format (Koranne and Koranne, 2011) which can be directly read by `CIT-Lasso` (He et al., 2026). Note that due to the block-diagonal partitioning in equation (1), this bundle of "pre-processed" LD files are typically dozens of gigabytes in size, and contain no individual level data. We emphatise this last point to invite researchers to share their results as they may be used by other studies on similar populations.

References

T. Berisa and J. K. Pickrell. Approximately independent linkage disequilibrium blocks in human populations. *Bioinformatics*, 32(2): 283, 2016.

Z. Chen, Z. He, B. B. Chu, J. Gu, T. Morrison, C. Sabatti, and E. Candès. Controlled Variable Selection from Summary Statistics Only? A Solution via GhostKnockoffs and Penalized Regression. *arXiv preprint arXiv:2402.12724*, 2024.

B. B. Chu, J. Gu, Z. Chen, T. Morrison, E. Candes, Z. He, and C. Sabatti. Second-order group knockoffs with applications to genome-wide association studies. *Bioinformatics*, 40(10):btae580, 2024.

J. R. Gimenez and J. Zou. Improving the stability of the knockoff procedure: Multiple simultaneous knockoffs and entropy maximization. In *The 22nd International Conference on Artificial Intelligence and Statistics*, pages 2184–2192. PMLR, 2019.

Z. He, Y. Le Guen, L. Liu, J. Lee, S. Ma, A. C. Yang, X. Liu, J. Rutledge, P. M. Losada, B. Song, et al. Genome-wide analysis of common and rare variants via multiple knockoffs at biobank scale, with an application to Alzheimer disease genetics. *The American Journal of Human Genetics*, 108(12):2336–2353, 2021.

Z. He, B. Chu, J. Yang, J. Gu, Z. Chen, L. Liu, T. Morrison, M. E. Belloy, X. Qi, N. Hejazi, et al. CIT-Lasso: a scalable approach beyond guilty by association for identifying causal variants from genome-wide summary statistics. *Genome Biology*, 27(1):223, 2026.

L. Jiang, Z. Zheng, H. Fang, and J. Yang. A generalized linear mixed model association tool for biobank-scale data. *Nature genetics*, 53(11):1616–1621, 2021.

S. Koranne and S. Koranne. Hierarchical data format 5: HDF5. *Handbook of open source tools*, pages 191–200, 2011.

Pan-UKB team. Pan-UK Biobank. https://pan.ukbb.broadinstitute.org, 2020.

F. Privé. Optimal linkage disequilibrium splitting. *Bioinformatics*, 38(1):255–256, 2022.

M. Sesia, C. Sabatti, and E. J. Candès. Gene hunting with hidden markov model knockoffs. *Biometrika*, 106(1):1–18, 2019.

M. Sesia, E. Katsevich, S. Bates, E. Candès, and C. Sabatti. Multi-resolution localization of causal variants across the genome. *Nature communications*, 11(1):1093, 2020.

M. Sesia, S. Bates, E. Candès, J. Marchini, and C. Sabatti. False discovery rate control in genome-wide association studies with population structure. *Proceedings of the National Academy of Sciences*, 118(40):e2105841118, 2021.

C. Sudlow, J. Gallacher, N. Allen, V. Beral, P. Burton, J. Danesh, P. Downey, P. Elliott, J. Green, M. Landray, B. Liu, P. Matthews, G. Ong, J. Pell, A. Silman, A. Young, T. Sprosen, T. Peakman, and R. Collins. UK BioBank: an open access resource for identifying the causes of a wide range of complex diseases of middle and old age. *PLOS Medicine*, 12:e1001779, 2015.

H. Zhou, J. S. Sinsheimer, D. M. Bates, B. B. Chu, C. A. German, S. S. Ji, K. L. Keys, J. Kim, S. Ko, G. D. Mosher, et al. OpenMendel: a cooperative programming project for statistical genetics. *Human genetics*, 139:61–71, 2020.
